# Diseases of Elephants

**Published:** 1882-04

**Authors:** 


Diseases of Elephants.—We desire to call the attention of our readers to the article written by Mr. Arstenstall, on the birth of an elephant, which took place at Bridgeport, in January, 1882. The writer is competent to speak on this matter, as he presided at the birth of the only two elephants born in America.

There are at present in this country eighty-eight elephants belonging to the different traveling menageries and zoological gardens, the value ef which ranges from $2,000 to $10,000 each, and where so much money is invested by a few persons in one species of animal, it seems important that the diseases incidental to the animal should be made a study of by our veterinarians, and not be left as they are at present, with one or two exceptions, to the treatment of men who act as keepers in the menageries. In the last number of the *Veterinary Journal* is found a list of the diseases to which the elephant is subject. The list comprises

Fever.
Inflammation of the brain and its membranes.
Inflammation of the lining membrane of facial sinuses.
Apoplexy.
Skin eruption from exposure to the sun.
Conjunctivitis.
Opacity of the cornea, due to indigestion.
Ulceration and sloughing of the external ear.
Progressive mortification of the tail.
Vomiting.
Spasmodic colic.
Flatulent colic.
Enteritis.
Purging, due to parasites.
Rot.
Bloody urine.
Asthemia.
Tetanus.
Rheumatism.
Boils.
Sprains.
Whitlow of the feet. Cracked sole. Bruised sole.
Inflammation of the lungs.
Serious effects of cold.
Foot and mouth disease, seen in Afghanistan campaign.
Galls on back.
Charbon.
Sunstroke.

The magnitude of this list is sufficient to show how important it is that veterinary surgeons should give the matter great attention. No book on the elephant has been written since Gilchrist's "Diseases of the Elephant," published in 1841, but we are promised shortly a work by Mr. H. H. Cross, who has spent thirteen years in India, with a view to study the habits and diseases of this animal.

